# Supplementary material for: Kin17 promotes rDNA transcription, ribosomal biogenesis, and cortical lamination
Source: EMBO Rep. 2025 Jul 17;26(17):4283–311. doi: 10.1038/s44319-025-00524-3 (PMC12420808; doi:10.1038/s44319-025-00524-3)
Supplement: Supplementary file 5 — Expanded View Figures [file 44319_2025_524_MOESM5_ESM.pdf]

# Expanded View Figures

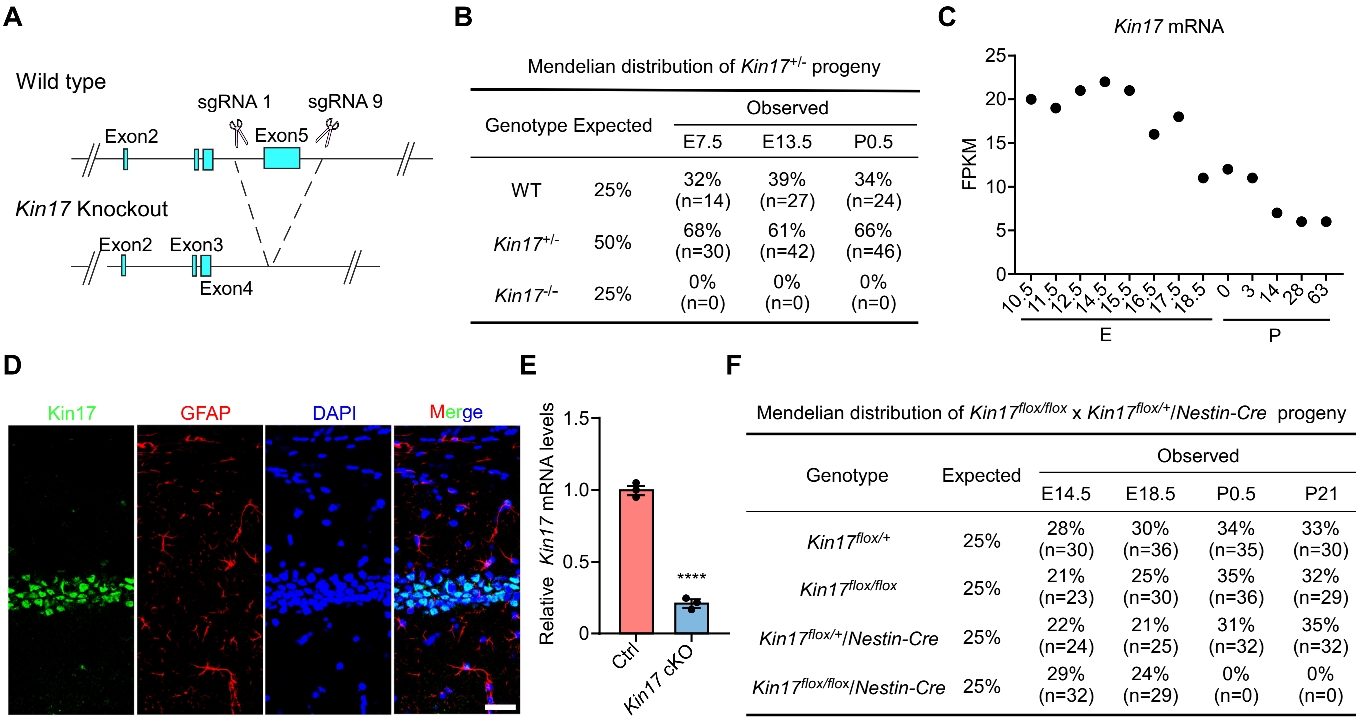


**Figure EV1. Mice with Kin17 depletion in neuronal progenitor cells show embryonic survival.**

(**A**) Generation of *Kin17* knockout mice. Schematic of the genomic target sites in *Kin17* locus. (**B**) Genotype distribution of embryos produced from mating of *Kin17*$^{+/-}$ mice at E7.5, E13.5, and P0.5. (**C**) Scatter plot showing Kin17 expression in mouse brains at embryonic and postnatal stages, based on data from the EMBL-EBI Expression Atlas. (**D**) Representative immunofluorescence images of Kin17 (green), GFAP (red), and DAPI (blue) in 3-month-old WT brain. Scale bar, 30 μm. (**E**) Kin17 mRNA levels in the cortex of control and *Kin17* cKO mice at E14.5, by qPCR ($P < 0.0001$). Data were normalized against β-actin ($n = 3$). (**F**) Genotype distribution of embryos and offspring from mating of *Kin17*$^{flox/flox}$ and *Kin17*$^{flox/+/Nestin\text{-}Cre}$ mice, assessed at E14.5, E18.5, P0.5, and P21. Statistical analysis was performed using two-tailed *t* test. Error bars denote the SEM. ****$P < 0.0001$.

 © The Author(s)

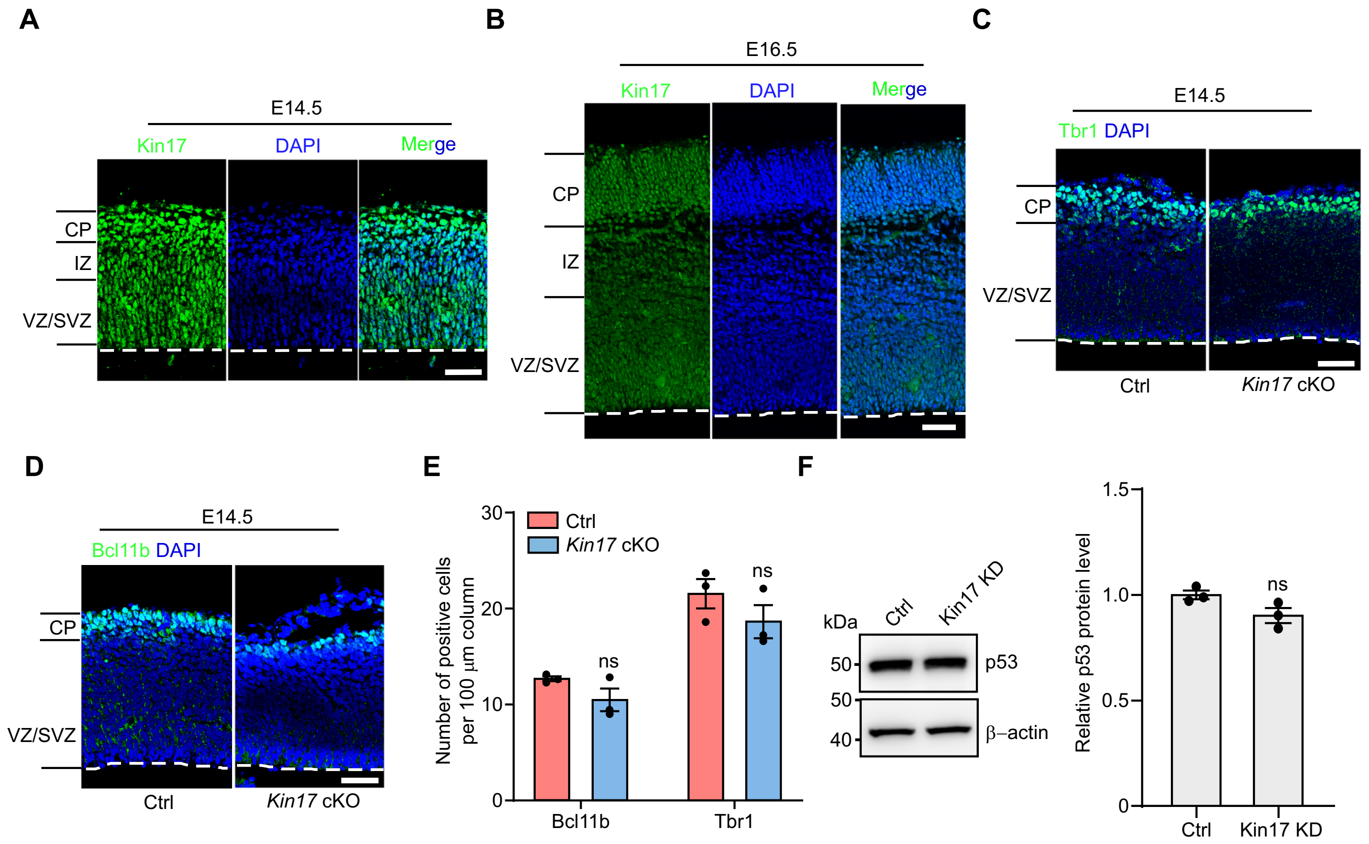


**Figure EV2. Kin17 depletion in NPCs leads to p53-independent apoptosis.**

(**A**, **B**) Representative immunofluorescence images of Kin17 (green) and DAPI (blue) in the cerebral cortex of WT mice at E14.5 (**A**) and E16.5 (**B**). Scale bar, 50 μm. (**C**, **D**) Representative immunofluorescence images of Tbr1 (green) and DAPI (blue) (**C**), of Bcl11b (green) and DAPI (blue) (**D**), in control and Kin17 cKO brain at E14.5. Scale bar, 50 μm. (**E**) Quantification of Tbr1 ($P = 0.1612$) or Bcl11b ($P = 0.2613$) positive cells in both control and Kin17 cKO brains ($n = 3$ per group). (**F**) p53 protein level in control and Kin17 KD 293T cells, by immunoblotting and densitometric analysis ($P = 0.0763$) ($n = 3$). β-actin was included as a loading control. Statistical analysis was performed using two-tailed $t$ test (**E**, **F**). Error bars denote the SEM. ns not significant.

© The Author(s) 

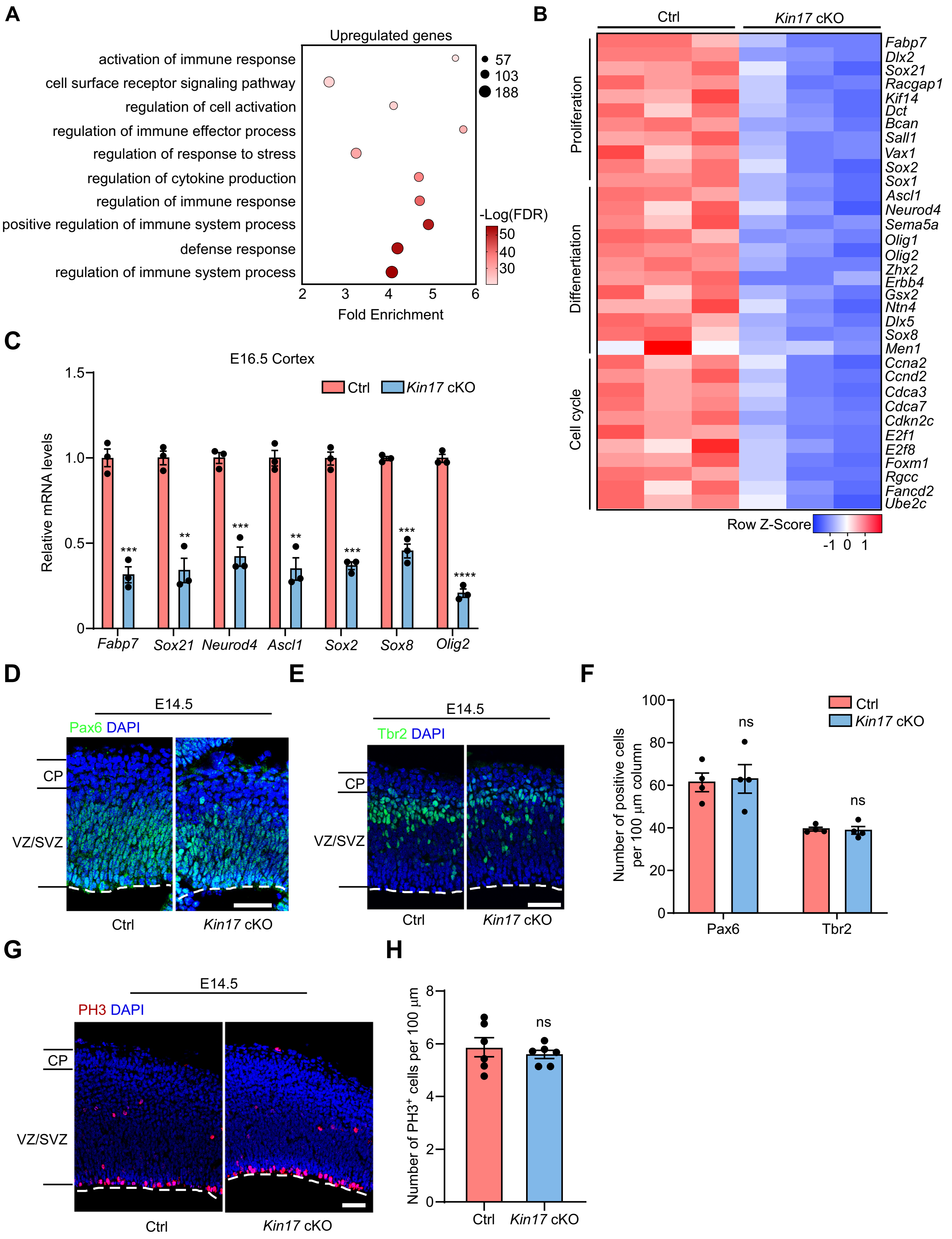
A
Upregulated genes
activation of immune response
cell surface receptor signaling pathway
regulation of cell activation
regulation of immune effector process
regulation of response to stress
regulation of cytokine production
regulation of immune response
positive regulation of immune system process
defense response
regulation of immune system process
57
103
188
-Log(FDR)
50
40
30
Fold Enrichment
B
Ctrl
Kin17 cKO
Proliferation
Differentiation
Cell cycle
Fabp7
Dlx2
Sox21
Racgap1
Kif14
Dct
Bcan
Sall1
Vax1
Sox2
Sox1
Ascl1
Neurod4
Sema5a
Olig1
Olig2
Zhx2
Erbb4
Gsx2
Ntn4
Dlx5
Sox8
Men1
Ccna2
Ccnd2
Cdca3
Cdca7
Cdkn2c
E2f1
E2f8
Foxm1
Rgcc
Fancd2
Ube2c
Row Z-Score
C
E16.5 Cortex
Ctrl
Kin17 cKO
Relative mRNA levels
Fabp7
Sox21
Neurod4
Ascl1
Sox2
Sox8
Olig2
D
E14.5
Pax6 DAPI
CP
VZ/SVZ
Ctrl
Kin17 cKO
E
E14.5
Tbr2 DAPI
CP
VZ/SVZ
Ctrl
Kin17 cKO
F
Ctrl
Kin17 cKO
Number of positive cells per 100 μm column
Pax6
Tbr2
ns
G
E14.5
PH3 DAPI
CP
VZ/SVZ
Ctrl
Kin17 cKO
H
Number of PH3+ cells per 100 μm
ns
Ctrl
Kin17 cKO

 © The Author(s)

**Figure EV3. Defective cortical lamination becomes evident at E16.5.**

(**A**) A bubble plot showing GO analysis of upregulated genes in the cerebral cortex of *Kin17* cKO versus control mice at E16.5. (**B**) A heatmap showing expressions of genes related to "proliferation", "differentiation", and "cell cycle" in the cerebral cortex of *Kin17* cKO versus control mice at E16.5 ($n = 3$ per group). (**C**) Levels of genes related to "proliferation" and "differentiation", by qPCR (Fabp7, $P = 0.0006$; Sox21, $P = 0.0013$; Neurod4, $P < 0.0009$; Ascl1, $P = 0.0013$; Sox2, $P = 0.0001$; Sox8, $P = 0.0002$; Olig2, $P < 0.0001$). Data were normalized against β-actin ($n = 3$). (**D**) Representative immunofluorescence images of Pax6 (green) and DAPI (blue) in the cerebral cortex of control and *Kin17* cKO mice at E16.5. Scale bar, 50 μm. (**E**) Representative immunofluorescence images of Tbr2 (green) and DAPI (blue) in the cerebral cortex of control and *Kin17* cKO mice at E16.5. Scale bar, 50 μm. (**F**) Quantitative analysis of Pax6 ($P = 0.778$) or Tbr2 ($P = 0.8618$) positive cells in control and Kin17 cKO cortices at E14.5 ($n = 3$ per group). (**G**) Representative immunofluorescence images of PH3 (red) and DAPI (blue) in the cerebral cortex of control and *Kin17* cKO mice at E14.5. Scale bars, 50 μm. (**H**) Quantitative analysis of PH3-positive cells in control and *Kin17* cKO mice ($P = 0.539$) ($n = 6$ per group). Statistical analysis was performed using two-tailed *t*-test. Error bars denote the SEM (**C**, **F**, **H**). **$P < 0.01$; ***$P < 0.001$; ****$P < 0.0001$; ns not significant.

© The Author(s)

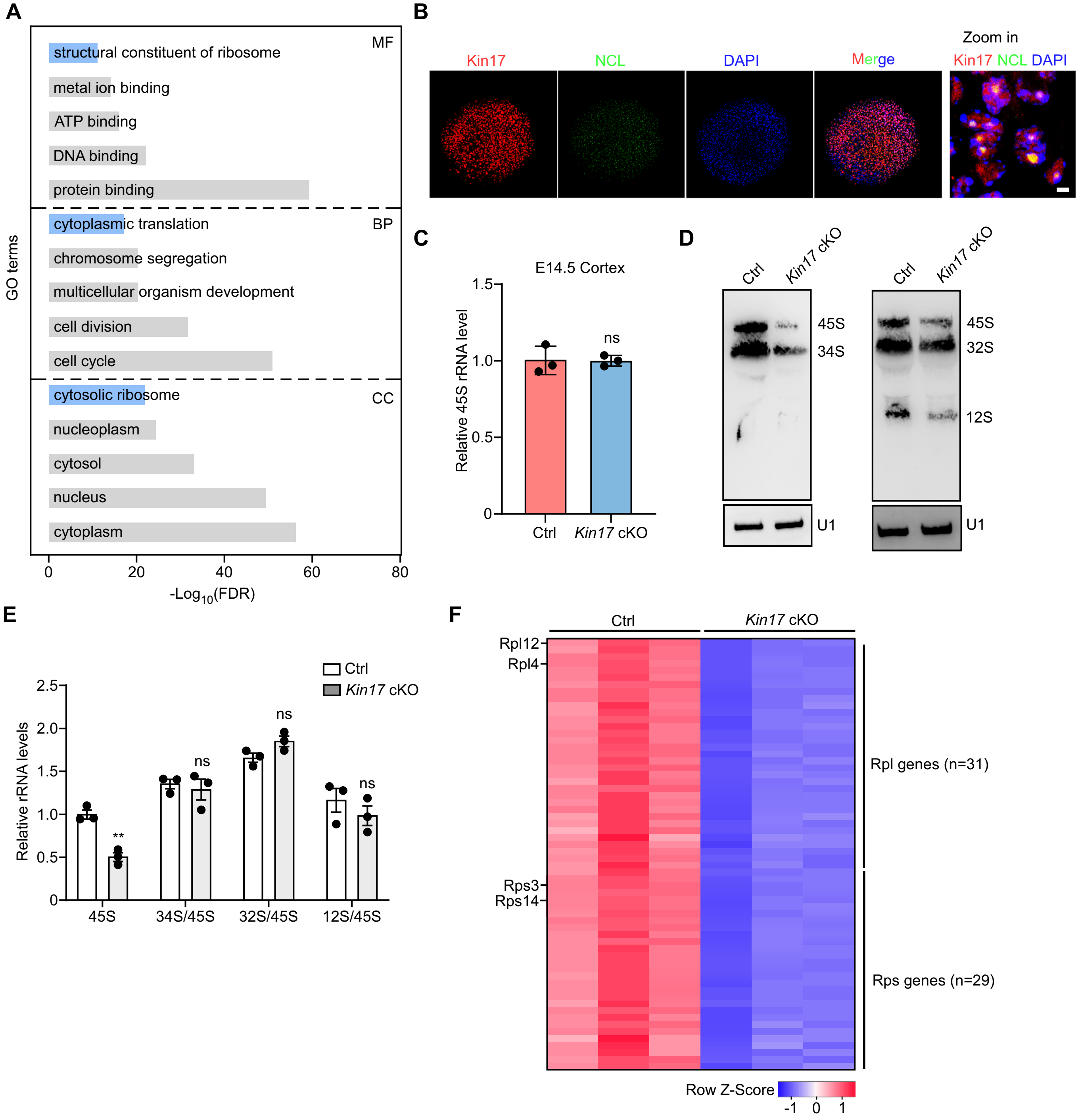


**Figure EV4. Kin17 has no impact on rRNA processing.**

(**A**) GO analysis of shared genes by molecular function (MF), biological process (BP) and cellular component (CC), highlighting enriched biological processes. (**B**) Representative immunofluorescence images of Kin17 (red), NCL (green), and DAPI (blue) in cultured neurospheres. Scale bar, 100 μm (left), 3 μm (right). (**C**) 45S rRNA level was determined in control and Kin17 cKO cortices at E14.5, by qPCR analysis ($P = 0.9689$) ($n = 3$). (**D, E**) Levels of 45S pre-rRNA, intermediate processed product 34S, 32S, and 12S in control and Kin17 cKO cortices were analyzed by northern blot (**D**) and densiometric analysis (**E**) ($n = 3$) (45S, $P = 0.0026$; 34S/45S, $P = 0.647$; 32S/45S, $P = 0.0722$; 12S/45S, $P = 0.3844$). U1 was included as a loading control. (**F**) A heatmap demonstrating differentially expressed ribosome protein genes in Kin17 cKO versus control neurospheres. Statistical analysis was performed using two-tailed Student's *t* test (**C**, **E**). Error bars denote the SEM. ns no significant.

 © The Author(s)

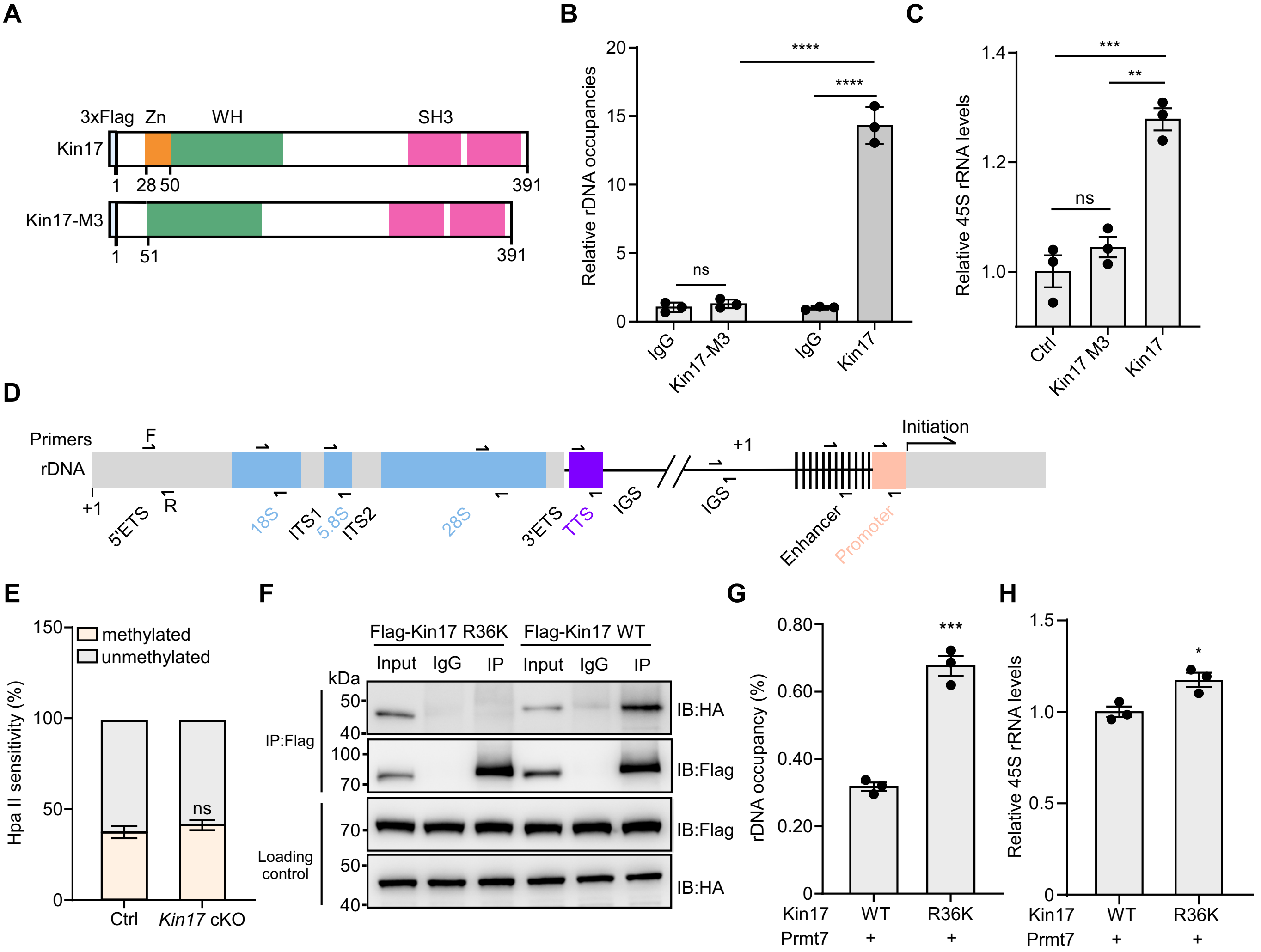


**Figure EV5. DNA binding domain is required for Kin17's binding to rDNA.**

(**A**) Schematic showing the generation of wild-type Kin17 (Kin17-WT) and the zinc finger domain-deleted mutant (Kin17-M3), each tagged with three Flag epitopes on the N-terminus. (**B**, **C**) N2a cells were transfected with either Kin17-M3 or Kin17-WT plasmids for expression. (**B**) Relative occupancy at the rDNA promoter was assessed by ChIP-qPCR using an anti-Flag antibody, followed by qPCR detection ($n = 3$) (Kin17-M3 versus IgG, $P = 0.3844$; Kin17 versus IgG, $P < 0.0001$; Kin17 versus Kin17-M3, $P < 0.0001$) (**C**) 45S pre-rRNA level was determined by qPCR-based analysis ($n = 3$). (Kin17-M3 versus control, $P = 0.6814$; Kin17 versus control, $P = 0.0005$; Kin17 versus Kin17-M3, $P = 0.0012$) (**D**) Schematic illustrating the primer design strategy for detecting the enhancer, promoter, 5′ external transcribed spacer (5′ETS), 18S, 28S, 5.8S, internal transcribed spacer (ITS), intergenic spacer (IGS), and transcription termination site (TTS) of rDNA. (**E**) Distribution of methylated and unmethylated rDNA in control or Kin17 cKO neurospheres ($n = 3$). (**F–H**) N2a cells were transfected with either HA-tagged WT Kin17 or R36K mutant, along with Flag tagged Prmt7 for expression. (**F**) The interaction of Kin17 and PRMT7 was assessed by co-IP using an anti-Flag antibody, followed by immunoblotting with an anti-HA antibody. (**G**) rDNA occupancy of Kin17 was determined by ChIP-qPCR analyses using an anti-HA antibody ($n = 3$) ($P = 0.0002$). (**H**) 45S pre-rRNA levels were determined by qPCR-based analyses ($n = 3$) ($P = 0.02$). Statistical analysis was performed using one-way ANOVA (**C**) with Bonferroni correction or two-tailed Student's *t* test (**B**, **E**, **G**, **H**). Error bars denote the SEM. $^{*}P < 0.05$, $^{**}P < 0.01$, $^{***}P < 0.001$; $^{****}P < 0.0001$; ns not significant.

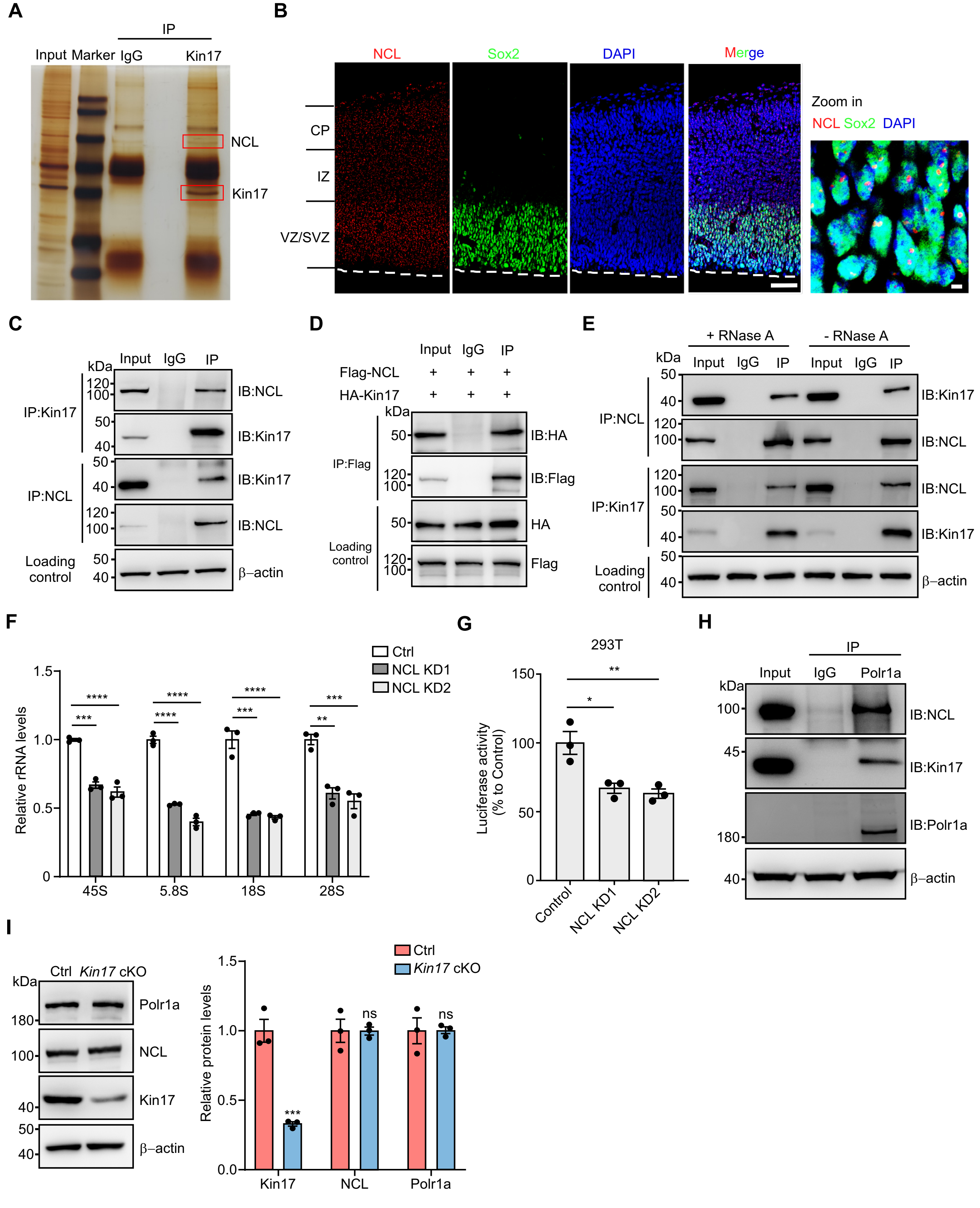
A
IP
Input Marker IgG Kin17
NCL
Kin17
B
NCL
Sox2
DAPI
Merge
CP
IZ
VZ/SVZ
Zoom in
NCL Sox2 DAPI
C
kDa Input IgG IP
IP:Kin17
IB:NCL
IB:Kin17
IP:NCL
IB:Kin17
IB:NCL
Loading control
β–actin
D
Input IgG IP
Flag-NCL
HA-Kin17
IP:Flag
IB:HA
IB:Flag
Loading control
HA
Flag
E
+ RNase A
- RNase A
Input IgG IP Input IgG IP
IP:NCL
IB:Kin17
IB:NCL
IP:Kin17
IB:NCL
IB:Kin17
Loading control
β–actin
F
Ctrl
NCL KD1
NCL KD2
Relative rRNA levels
45S
5.8S
18S
28S
G
293T
Luciferase activity (% to Control)
Control
NCL KD1
NCL KD2
H
IP
Input IgG Polr1a
IB:NCL
IB:Kin17
IB:Polr1a
β–actin
I
Ctrl Kin17 cKO
Polr1a
NCL
Kin17
β–actin
Ctrl
Kin17 cKO
Relative protein levels
Kin17
NCL
Polr1a

 © The Author(s)

**Figure EV6. Kin17-NCL interaction is required to promote rDNA transcription.**

(**A**) Kin17-associated proteins were separated by electrophoresis and visualized by silver staining. (**B**) Representative immunofluorescence images of NCL (red), Sox2 (green), and DAPI (blue) in the cerebral cortex of WT mice at E16.5. Scale bar, 50 μm (left), 3 μm (right). (**C**) The interaction of endogenous Kin17 and NCL in the cortex of WT mice at E16.5, assessed by co-IP using an anti-Kin17 antibody followed by immunoblotting detection with an anti-NCL antibody, and reciprocally by co-IP with an anti-NCL antibody followed by immunoblotting detection with an anti-Kin17 antibody. (**D**). The interaction of exogenous HA-Kin17 and Flag-NCL in 293 T cells transfected with HA-Kin17 and Flag-NCL for expression, determined by co-IP with an anti-Flag antibody and immunoblotting detection with an anti-HA antibody ($n = 3$). (**E**) Neurosphere lysates were treated with either RNase A or a control solvent, followed by co-IP using an anti-NCL antibody. The NCL-associated Kin17 was detected by immunoblotting with an anti-Kin17 antibody. Reciprocally, co-IP was performed using an anti-Kin17 antibody, and the Kin17-associated NCL was similarly detected by immunoblotting with an anti-NCL antibody. (**F**) The levels of rRNA (45S, 5.8S, 18S, and 28S) in NCL KD neurospheres, by qPCR ($n = 3$ per group) (45S: NCL KD1, $P = 0.0002$; NCL KD2, $P < 0.0001$; 5.8S: NCL KD1, $P < 0.0001$; NCL KD2, $P < 0.0001$; 18S: NCL KD1, $P = 0.0001$; NCL KD2, $P < 0.0001$; 28S: NCL KD1, $P < 0.0017$; NCL KD2, $P < 0.0008$). Data were normalized against U1 snRNA. (**G**) rDNA promoter reporter plasmid was transfected into control or NCL KD 293 T cells. Luciferase activity was determined by Dual-Luciferase reporter assay ($n = 3$ per group). (**H**) The interaction of Polr1a with NCL or Kin17 in neurospheres, determined by co-IP with an anti-Polr1a antibody, followed by immunoblotting detection with an anti-NCL antibody or an anti-Kin17 antibody ($n = 3$ per group). (**I**) Polr1a and NCL expression in control and Kin17 cKO neurospheres, by immunoblotting and densitometry ($n = 3$ per group) (Kin17, $P = 0.0013$; NCL, $P = 0.9818$; Polr1a, $P = 0.9477$). Statistical analysis was performed using one-way ANOVA with Bonferroni correction (**F**, **G**) or two-tailed Student's $t$ test (I). Error bars denote the SEM. *$P < 0.05$; **$P < 0.01$; ***$P < 0.001$; ****$P < 0.0001$; ns not significant.

© The Author(s)
